# Secrecy in Venereal Diseases

**Published:** 1920-01-24

**Authors:** 


### SECRECY IN VENEREAL DISEASES.

A good deal has been written in the Press during the past week on the subject of medical evidence in Courts of Law. It is an old question, but it takes new interest for the social reformer in this particular case, because it was concerned with venereal disease, the prevention and cure of which is a matter of such great importance.

The Judge compelled a medical witness to give evidence that he had treated a person for the disease, and over-ruled the witness's claim of the privilege of secrecy under regulations made in pursuance of the Public Health Acts. There is no doubt that, while the Judge was within his powers in insisting upon the principle of the law of evidence, the medical profession is strongly opposed to disclosing the confidences of their patients, while social reformers have fears that the whole Government scheme for exterminating venereal disease may be jeopardised if secrecy cannot be maintained with the assistance of the courts. Yet in the case which started the discussion one of the grounds of the petitioner's appeal was cruelty by communication of the disease, and without medical evidence proof could not have been maintained. There are, therefore, the two considerations—justice and prevention of foul disease. If secrecy is found to be an essential part of a successful campaign, is it not possible for justice to be done by permitting medical evidence to be given in secret where the just settlement of a case necessitates such evidence? There is already a quite sufficient amount of unsavoury material emitted from the Divorce Courts.

### Confidence Not Disclosed.

Much of the controversy on this case, however, has been off the mark. The medical evidence was called by the petitioner to prove that she was suffering from a disease communicated to her. She therefore invited the medical man to make known the secret, and it is difficult to see any objection to that course on common-sense grounds. It was not disclosing a confidence of an unwilling party, and, if it involved the exposure of the respondent, that was an incidental result which has no bearing whatever on the subject of respecting confidences. It would have been grossly unjust to the petitioner if that consideration had been allowed to stand in her way.

